# PRECARIOUS PATHWAYS INTO RETIREMENT AND NEW RISKS FOR GENDERED ECONOMIC EXCLUSION IN SWEDEN, 1990-2015

**DOI:** 10.1093/geroni/igz038.478

**Published:** 2019-11-08

**Authors:** Andreas Motel-Klingebiel, Susanne Kelfve

**Affiliations:** Linkoping University, Linkoping University, Sweden, Sweden

## Abstract

The ability and disposition of ageing people to maintain their labour market activity and/or to retire from work structurally depend on pension systems, activation policies, ageism, changing for labor demand and economic shifts. Structural conditions are changing, but social change does not mature homogeneously and neither do the institutional shifts induced by it. Gains in opportunities and resources do not benefit all people, groups and even societies in the same way. Changes increase insecurities and life course inhomogeneity, create unequally distributed challenges and show asynchrony in shifts and outcomes. They generate new precarity in ageing and socially structured risks for exclusion in work and retirement and refer to existing later life inequalities by cohort, gender, region, education, class and ethnicity. From this perspective of ageing and social change, the paper deals with shifts in late work and retirement patterns and later-life outcomes under changing institutional conditions, focusing on gendered risks for economic exclusion and later life precarity in Sweden. Swedish registry data comprising individual work and health histories as well as employer, regional and neighborhood information on the total population 50+ ever living in Sweden 1990-2015 is used in a cohort sequential perspective. Analyses focus on gender inequalities and concentrate on occupational activities, retirement transitions and pension revenues under changing social conditions. Models find increasingly heterogeneous preretirement and transition patterns, new gender gaps and increasing risks of economic exclusion in retirement with disadvantaged groups as forerunners in overall relative declines in later-life economic positions.

